# Supplementary material for: Content-rich biological network constructed by mining PubMed abstracts
Source: BMC Bioinformatics. 2004 Oct 8;5:147. doi: 10.1186/1471-2105-5-147 (PMC528731; doi:10.1186/1471-2105-5-147)
Supplement: Additional File 2 — The original results of the above study (non-essential files are deleted to keep the file size under the limit set by BMC bioinformatics). [file 1471-2105-5-147-S2.bz2 › chilibotAdditionalFile2/dip20/127ID8609467E471/html/P_N.html]

 


 **P** and **N** 
  
Found 149323 abstracts in PubMed, retrieved 20.  
 

 What does Google say? 
 PDF only 
| .edu only 

---

**Interactive relationship** (e.g. stimulation, inhibition, etc)

**Neutral relationship**- Subjects with diabetes mellitus  **n**  = 87 had higher concentrations of HDL associated PAF AH 0.50 0.23 g mL,  **P** < ; 0.01 and lower concentrations of nonHDL associated PAF AH 0.86 0.46 vs 1.15 0.51 g mL  **P** < ; 0.01 than controls.  Ref: 12867534 J Lipid ResJ Lipid Res,
- Subjects with hyperlipidemia  **n** =69 had higher HDL associated PAF AH concentrations than controls 0.61 0.32 vs 0.14 0.07 g mL,  **P**  &lt.  Ref: 12867534 J Lipid ResJ Lipid Res,
- In healthy subjects plasma total PAF AH concentration was greater in men than in women, 1.38 0.57  **n** =69 vs 1.09 0.45 g mL  **n** =29, mean SD,  **P** < ; 0.01, and was positively correlated with PAF AH activity and with plasma total cholesterol, triacylglycerol, low density lipoprotein LDL cholesterol and apo B concentrations all  **P**  &lt.  Ref: 12867534 J Lipid ResJ Lipid Res,

**Non-interactive relationship** (e.g. studied together, co-existance, homology, etc.)

- **P** < ; 0.0001,  **n** =43, the concentration of apo B at D>1.044 g ml correlated strongly with both corrected results r=0.978.  Ref: 12867279 Clin Chim Acta, 2003
- Heterozgotes for the common mutation at position 279 V279F had lower total PAF AH concentrations 0.79 0.24 g mL,  **n** =37 than subjects with the wild type 1.61 0.44 g mL,  **P** < ; 0.01.  Ref: 12867534 J Lipid ResJ Lipid Res, 2003
- The median number of days with positive blood cultures was 2 for the MRSA  **n**  = 16 and 1 for the MSSA  **n**  = 18 patients  **P**  = 0.04.  Ref: 12867833 Pediatr Infect Dis JPediatr Infect Dis J, 2003
- Enrichment of nutrients such as nitrogen  **N** , phosphorous  **P** , potassium K in biosolids was recorded in 6.2% w w, 3.1% w w and 0.15% w w from its initial values of 4.8% w w, 2.0% w w and 0.08% w w respectively after 10 days of fungal treatment.  Ref: 12867323 Water Res, 2003
